# Combination of health care service use and the relation to demographic and socioeconomic factors for patients with musculoskeletal disorders: a descriptive cohort study

**DOI:** 10.1186/s12913-023-09852-3

**Published:** 2023-08-14

**Authors:** Olav Amundsen, Tron Anders Moger, Jon Helgheim Holte, Silje Bjørnsen Haavaag, Line Kildal Bragstad, Ragnhild Hellesø, Trond Tjerbo, Nina Køpke Vøllestad

**Affiliations:** 1 Dept. for Interdisciplinary Health Sciences, Institute of Health and Society, University of Oslo, Oslo, Norway; 2 Dept. of Health Management and Health Economics, Institute of Health and Society, University of Oslo, Oslo, Norway; 3 Dept. of Public Health Science, Institute of Health and Society, University of Oslo, Oslo, Norway

**Keywords:** Health care utilization, Musculoskeletal, Register-based research

## Abstract

**Background:**

Patients with musculoskeletal disorders (MSDs) access health care in different ways. Despite the high prevalence and significant costs, we know little about the different ways patients use health care. We aim to fill this gap by identifying which combinations of health care services patients use for new MSDs, and its relation to clinical characteristics, demographic and socioeconomic factors, long-term use and costs, and discuss what the implications of this variation are.

**Methods:**

The study combines Norwegian registers on health care use, diagnoses, comorbidities, demographic and socioeconomic factors. Patients (≥ 18 years) are included by their first health consultation for MSD in 2013–2015. Latent class analysis (LCA) with count data of first year consultations for General Practitioners (GPs), hospital consultants, physiotherapists and chiropractors are used to identify combinations of health care use. Long-term high-cost patients are defined as total cost year 1–5 above 95^th^ percentile (≥ 3 744€).

**Results:**

We identified seven latent classes: 1: GP, low use; 2: GP, high use; 3: GP and hospital; 4: GP and physiotherapy, low use; 5: GP, hospital and physiotherapy, high use; 6: Chiropractor, low use; 7: GP and chiropractor, high use. Median first year health care contacts varied between classes from 1–30 and costs from 20€-838€. Eighty-seven percent belonged to class 1, 4 or 6, characterised by few consultations and treatment in primary care. Classes with high first year use were characterised by higher age, lower education and more comorbidities and were overrepresented among the long-term high-cost users.

**Conclusion:**

There was a large variation in first year health care service use, and we identified seven latent classes based on frequency of consultations. A small proportion of patients accounted for a high proportion of total resource use. This can indicate the potential for more efficient resource use. However, the effect of demographic and socioeconomic variables for determining combinations of service use can be interpreted as the health care system transforming unobserved patient needs into variations in use. These findings contribute to the understanding of clinical pathways and can help in the planning of future care, reduction in disparities and improvement in health outcomes for patients with MSDs.

**Supplementary Information:**

The online version contains supplementary material available at 10.1186/s12913-023-09852-3.

## Introduction

Musculoskeletal disorders (MSDs) are among the most prevalent conditions globally and in Norway [1, 2]. Chronic MSDs are common in the general population and the prevalence increases with age [1]. MSDs are the leading cause for years lived with disability in Norway and accounts for one third of all sickness benefits, 30% of all disability pensions and 9% of all direct health care costs [3, 4]. The annual total cost of MSDs in Norway was estimated at approximately 22 billion Euros in 2015 [3]. This makes MSDs both the most prevalent and costly diagnosis group [4], indicating a major challenge that puts significant pressure on societal resources.

The lack of a single intervention or cure for most MSDs has led to a large variation of care for patients. Some of the main challenges in the management of MSDs are the increasing use of treatments considered discordant with guidelines for MSDs [5, 6], high risk of overuse of low-value care for both examinations, surgical and non-surgical interventions and the underuse of cheaper and equally effective interventions [7–10]. Many MSDs share common risk profiles and prognostic factors for pain and disability [11, 12] and a systematic review assessing guidelines for neck pain, low back pain, osteoarthritis and rotator cuff disorders found that the guidelines have a high consensus irrespective of body regions [11, 13]. Guidelines recommend that most non-traumatic MSDs should be managed in primary care, be patient-centred and that first-line management should consist of education, advice and addressing exercise and physical activity. Non-surgical care should be offered prior to surgery unless specifically indicated [13, 14]. If real-life practice was in line with the guidelines, we would expect that most patients with non-specific MSDs (disorders characterised by symptom location, i.e. low back pain, neck pain, rotator cuff related pain) could be managed with low health care use in primary health care with an emphasis on supporting self-management. We would also expect that specific MSD disorders (i.e. fractures, knee ligament injuries) might have a more distinct care pattern with high initial health care use and use of specialist health care for a short period.

Despite this, some patients with MSDs have a high health care use over long time periods [15], indicating that certain patient groups are not able to manage their MSDs with low and short-term resource usage. Patients with lower socioeconomic status and immigrant background have higher burden of MSDs and other chronic diseases, higher risk of poor outcomes, poorer general and musculoskeletal health [16–22] and struggle more with self-managing health conditions [20, 23, 24]. Demographic and socioeconomic factors are also associated with total health care use [15, 25]**.** Given the high prevalence and large variation in health care use and outcomes, there is a growing interest in how to best organise care for MSDs [26]. It has been pointed out that health care systems are mainly organised into silos handling acute and episodic conditions, and might not be equipped to meet the requirements of those with chronic health problems [27]. Chronic conditions, such as many MSDs, might require ongoing management over longer periods of time for some patients and often need coordinated care from several health care professionals in different settings [28]. There are a lack of studies that examine how people with MSDs utilize combinations of different health care services in their illness management, and how health care use is related to demographic and socioeconomic factors and diagnoses.

A first step in improving care would be to investigate how patients with MSDs uses different health care services in combination in current practice. This information can help inform health care personnel, patients and stakeholders when planning and prioritising care for MSDs. Our objective was to identify which combinations of health care services patients use for a new MSD and explore variations between different combinations of clinical characteristics, demographic and socioeconomic factors and long-term use and costs. Our hypothesis was that there would be a large variation in service use and combinations for patients with MSDs, and that the service use would be related to different demographic and socioeconomic profiles and different diagnoses.

## Methods

### Design and setting

This study is a descriptive cohort study conducted as part of the project Innovations in use Of REGistry data (INOREG) at the Institute of Health and Society, University of Oslo. The project combines several data registries from 2008 to 2020 to create a cohort with health care use, demographic and socioeconomic factors and outcomes for chronic diseases in Norway, where MSDs are used as a case representing non-specific disorders. The present study uses national registers with primary and specialist health care data, capturing the entire use of public health care in Norway. The study includes patients that were registered as residents in Oslo or Trondheim at any point between 2008–2020. The reporting of this study adheres to the Strengthening the Reporting of Observational Studies in Epidemiology (STROBE) checklist for observational studies with the REporting of studies Conducted using Observational Routinely-collected Data (RECORD) extension [29, 30].

This study utilizes data on health care use for MSDs in the Norwegian public health care service. Norway has a national universal health care system with an overarching goal of equal access to services for all inhabitants [31]. The main providers for MSD-care in Norway are General practitioners (GPs), hospital services, physiotherapists and chiropractors. GPs and physiotherapy are part of municipal primary care, hospital services secondary care and chiropractors function as private practitioners.

GPs and physiotherapists usually have contracts with the municipalities where the GPs receive capitation and physiotherapists a fixed amount for running the service within the municipality. The practitioners are self-employed. In addition to the basic funding, their services are partly funded by out-of-pocket payments and partly as fee-for-service from The Norwegian Health Economics Administration (HELFO). These fee-for-service reimbursements are registered in the registry The Control and reimbursement of health care claims (KUHR) together with diagnoses. This allows identification of patient consultations in primary care that is used in this study. Costs for consultations with GPs and physiotherapists are set by a national standard, with the cost per consultations shared approximately equally between out-of-pocket payment and fee-for-service reimbursement from HELFO.

There are cost-sharing ceilings with a maximum limit for out-of-pocket payment costs to protect individuals from high health care expenditures. At the time of inclusion (2013–2015) there was one ceiling for treatment by physicians and psychologists, some medicines, diagnostic tests and transportation expenses related to examination and treatment and one ceiling for physiotherapy and accommodation at rehabilitation centers and treatment (approximately 175–220€ each). When this cost ceiling is reached, HELFO covers the patient’s out-of-pocket payment for the health services as well [31].

Chiropractors are not included as part of the municipal health care service but function as private practitioners. HELFO covers a fee-for-service (6.5€ per consultation), and the chiropractors can set their own price for patients’ out-of-pocket payment. The fee-for-service from HELFO covers approximately 10–15% of the full price [32]. Out-of-pocket expenses are not covered by the cost-sharing ceiling for chiropractors. At the time of inclusion, HELFO covered fourteen consultations per year, meaning that data on chiropractor consultations that exceeds this limit are not available. This limit was removed in 2017 [33]. The financing of the chiropractors’ services is very different to the other services, and it is not possible to directly compare costs between chiropractors and the other services.

Additionally, physiotherapists in private practice and other groups such as naprapaths and osteopaths work fully in the private market, and do not receive fee-for-service payment from HELFO and are hence not registered in KUHR.

Inpatient and outpatient hospital care is provided by hospital trusts owned by regional health authorities. Inpatient care in hospitals is fully covered for patients, while outpatient care has an out-of-pocket fee of approximately 33€, unless the patient has reached the cost-sharing ceiling. The hospitals are funded by block grants and activity-based funding based on the Nordic diagnosis-related groups (DRG) system to classify patients. The total hospital funding is split almost equally between the two funding sources. All hospital consultations, both inpatient and outpatient, are registered in the Norwegian Patient Registry (NPR) together with diagnosis, DRG and a cost weight per DRG. This includes consultations with all health care personnel. These data allow identification of all public specialist health care use and can be used to calculate the costs for each consultations.

Patients typically make their initial contact with the health care system through their GP, who functions as a gatekeeper to specialist care. At the time of inclusion, the patient also needed a referral to physiotherapy from their GP. Direct access to physiotherapy was established in 2018. Physiotherapists with a master’s degree in treatment of MSDs (manual therapists) and chiropractors have had direct access and the right to refer to specialist health care, radiological examinations and prescribe sick leave up to 12 weeks for MSDs since 2008.

### Data sources and variables

This study used data from the national registry KUHR for identifying patients with MSDs and capture primary health care utilization. This allows calculation of use and costs for GPs, physiotherapists and chiropractors. The NPR was used to capture specialist health care use and to calculate the associated costs. Statistics Norway was used for demographic and socioeconomic factors and Statistics Norway's historical events database (FD-trygd) for information on social services, sick leave and disability benefits. Patients that died during the investigation period were identified from the Norwegian Cause of Death Registry. The chosen registries provide a complete overview over public health care use and social service use, meaning there is no loss to follow-up due to lack of reporting. Registry data from 2008–2020 were matched on an individual level by using pseudonymized national ID-numbers. A detailed list of data sources, variables and their definitions used in this study are presented in Table 1. Categorizations and definitions are based on definitions by Statistics Norway if available or created by the research group based on common practice.

**Table 1:** Data sources, variables and variable definitions

Source	Variable	Definition
KUHR	Index date	First registered date with a MSD-related health care consultation
	Diagnosis	International Classification of Primary Care, 2nd edition (ICPC-2) codes within chapter “L; Musculoskeletal” was used to categorise consultations and costs related to MSDs and to other conditions. The first MSD-related diagnosis were registered and categorised: Neck pain (L01, L83), back pain (L02, L03, L84-L86), upper extremity (L08-L12, L92, L93), hip/thigh/knee (L13-L15), ankle/foot (L16, L17), widespread pain/fibromyalgia (L18), fracture or joint-/ligament injury (L72-L80, L96), other soft tissue (L87), unspecified/other MSDs (all other L-diagnosis). Patients registered with the specific codes L70 (Infection of musculoskeletal system), L71 (Malignant neoplasm musculoskeletal), L88 (Rheumatoid/seropositive arthritis and L97 (Neoplasm musculoskeletal benign/unspecific) were excluded
	Comorbidity	A previously adapted comorbidity index from GP-diagnosis based on ICPC-2 diagnosis, which have been validated to be used as an adjustment variable in epidemiological research in primary care databases [34]. This is a comorbidity index based on primary care data with eighteen selected diagnoses and the individual patient are assigned an index score based on number of diagnoses that can be identified. The comorbidity index were dichotomised into 0–1/2 or more
	Frequency of consultation	Frequency of consultations for GP, physiotherapy, chiropractors and contract specialists with and without an MSD-diagnosis. Consultation was defined as a health care contact with a fee indicating a face-to-face/video-consultation individually or group based and does not include fees that indicate a simple communication, prescription writing or administrative work. This approach has shown high validity for the GP-service [35]. Included fees: GP: 2ad, 2ak, 2ae, 2ed Physiotherapy: A2a-f, A3a-b, A8a, A9a, A1a, A1d, c34 Chiropractors: K1, K2
	Health care reimbursement cost	All reimbursement fees for contacts with an MSD-diagnosis. Aggregated cost per year per service and total cost per year. Costs are calculated in Norwegian currency but written in text as Euro (1€ = ~ 11NOK)
NPR	Diagnosis	ICD-10 codes [36] within chapter “Diseases of the musculoskeletal system and connective tissue” was used to categorise consultations and costs related to MSDs and to other conditions. Patients registered with the specific codes related to infections (M00, M01), malignant disease (M86) and inflammatory rheumatic disease (M05-M08, M13, M30-M33, M35, M45, M46) were excluded. Codes within chapter “Injury, poisoning and certain other consequences of external causes” related to MSDs was included. This include S32-34, S40-S99 and T08-T13
	Frequency of consultations	Frequency of consultations after index date registered as outpatient contact or inpatient stay with and without an MSD-diagnosis. This includes consultations with all health care personnel
	Health care cost related to DRGs	Cost weight of DRG (corrected version) per MSD-related consultation multiplied with cost of 1 DRG for the specific year. Cost of 1 DRG have increased from 39,447 NOK in 2013 to 45,808 NOK in 2020. Costs are calculated in Norwegian currency but written in text as Euro (1€ = ~ 11NOK)
Statistics Norway	Age	Age in years at index date
	Gender	Male/female
	Education	Highest registered education. Categorised: 13 years or less/more than 13 years
	Income	Income registered the year before inclusion. Presented as median income,
	Immigrant background	Categorised: No immigration background or Any immigration background (Includes: first generation immigrant, Norwegian-born second generation immigrant, one foreign parent, born outside Norway from Norwegian parents)
FD-Trygd	Work status	Work status. Categorised as: Employed/self-employed or not registered as employed
	Sick leave	Number of days registered with sick leave within 365 days from index date
	Disability pension	If the patient is registered on disability pension prior to inclusion
Norwegian Cause of Death Registry	Date of death	Date of death were used to exclude patients that died during the first year of follow up and exclude patients that died during the first five years from the long-term cost analysis

*Abbreviations*: *KUHR* The Control and reimbursement of health care claims. *NPR* Norwegian Patient Registry. *MSD* Musculoskeletal disorder. *GP*: General practitioner. *ICPC-2* International Classification of Primary Care, 2nd version. *ICD-10* International Statistical Classification of Diseases and Related Health Problems 10th Revision. *DRG* Nordic diagnosis-related groups

### Sample selection

In this study, we included patients with an MSD-related health care contact (ICPC-2 Chapter L) in 2013–2015 and no history of MSD-contacts the previous three years. Prior studies have indicated that a three-year wash-out period is optimal for common MSDs to exclude ongoing disorders and identify new ones [37, 38]. We used a starting point from 2013–2015 as this was the earliest period where data from all health care services was complete. The data for the GP-service is complete from 2008, allowing us to use the period prior to 2013 as a washout-period. This ensured a minimum follow-up time of five years for each patient, allowing us to assess long term costs and use. The first MSD-related contact in 2013–2015 served as an index date, regardless of which health care professional registered the first contact. Patients below 18 years or that died within one year after the index date were excluded. Patients with MSD-diagnoses related to infection, tumor or inflammatory rheumatic diseases (ICPC-2 codes: L70, L71, L88 or L97. ICD-10 codes M00, M01, M05-08, M12, M13, M30-33, M35, M45, M46 M60, M65 and M71 and M86) were excluded as the present study focused on symptom-based diagnoses. We also created a cohort by the same criteria from 2018–2019 to compare our findings to a separate and more recent cohort, established after relevant legislation changes MSDs [33, 39].

### Statistical analysis

We used latent class analysis (LCA) to explore the various combinations of health care services used by patients with MSDs. We used data with aggregated number of consultations related to MSDs per service within 365 days after the index date for GP, hospital services, physiotherapy and chiropractors in the LCA [40]. Additional services such as contract specialists and rehabilitation units were considered for inclusion in the model but were excluded due to limited usage (1.9% and 0.07%, respectively). We started with a one-class model and added an additional class each time. Model fit was examined by using the Bayesian information criterion (BIC), Likelihood Ratio (LR), average posterior probability (APP), interpretability and subjective considerations as to whether the classes were meaningful. Likelihood-ratio tests were used to compare each model to the previous model with one less class [40–42]. The LCA was also conducted in a separate cohort included from 2018–2019 to check for stability of the model. There was a relative reduction in BIC and significant likelihood ratio tests when comparing each of the new models containing one more class to the previous. Models with more than 13 classes failed to converge. From the model with seven classes and onwards, there were minor changes in BIC and Likelihood Ratio (< 2%). The APP for all classes was above the cut-off value of 0.80 for all models, except the model with eleven subclasses. When the models have similar fit statistics, it is important to consider whether the added subgroup makes conceptual sense and increases interpretability [40].

We chose the 7-class model, as this was the last model that had more than a 2% reduction of BIC and added a new relevant class compared to the 6-class model by dividing chiropractor use into low and high use. The 7-class model was also reproduced in the 2018–2019 cohort with very similar classes (supplementary 3), indicating model stability. Fit statistics and descriptions of classes for all models are included in the supplementary (supplementary 1) to ensure transparency in reporting. The classes with different combinations of health care use are illustrated with a bar graph that shows median and the interquartile range (IQR) for consultations for each health care service. Class descriptions are based on how the class is distinct from the other classes in the same model. The classes are first described by the type of health care service that are used. When there is more than one class with the same service use, it is further described by being low or high use of primary care services relatively to the other class in the same model.

We conducted an additional subgroup comparison in two of the classes (class 2 and 5, see below), as approximately 50% used hospital services and 50% used only primary care services in these classes.

The health care use is further elaborated on for each class by showing the proportion using each service, the total health care consultations and reimbursement costs, and health care use for other diagnoses than MSDs. The observed age, gender, income, education, immigration background, comorbidity, sick leave during the first year and diagnoses in each class are presented to allow comparison of demographic and socioeconomic factors between classes. Means and standard deviation (SD) are used for variables that follow a normal distribution, median and IQR are used for variables with a skewed distribution and percentages are used for binary variables. Data on diagnoses are presented as a stacked bar graph illustrating the total prevalence and the prevalence within each class.

We also assessed which patients ends up as long-term high-cost users and which patients have no costs after the first year. For this analysis we excluded patients that died during the follow-up period (9 729 patients), and patients with index date after 12^th^ March 2015 (51 249 patients) as their 5^th^ year health care use would likely be affected by the COVID-19 pandemic and the lockdown that started 12^th^ March 2020. We assessed different cut-off points for high-cost users, as there are large differences in how this is defined in the previous literature [43–47]. For the results section we have defined long-term high-cost users as patients with a total cost above the 95^th^ percentile for the first five years combined (3 744€). We also present a sensitivity analysis with cut-offs above the 90^th^ percentile (1 728€) and the 99^th^ percentile (12 603€) in the supplementary (supplementary 4). We also studied who the high-cost users were in the period from the second to the fifth year and have included these data in the supplementary as a sensitivity analysis. We assessed how many patients had no future health care costs after the first year in the results chapter, and how many had no costs after the second year as a sensitivity analysis. Stata (version 17.0) was used for all data management and analysis.

## Results

**Fig. 1:**
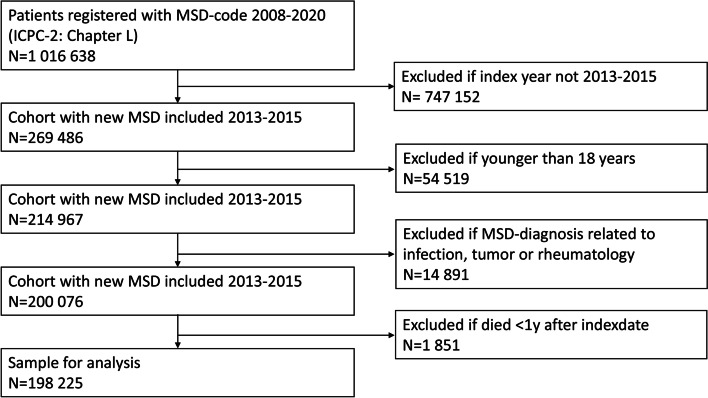
Flow chart of sample selection process. MSD = Musculoskeletal Disorder

The preliminary database included 1 016 638 patients registered with an MSD-diagnosis between 2008 and 2020. After excluding patients based on index year, age, diagnosis and death, the final sample consisted of 198 225. A flowchart for sample selection is shown in Fig. 1.

### Description of the full sample

The total sample had a mean age of 43.6 (SD 18.0) years, with equal proportions of male and female patients. There was a median of two health care consultations for MSDs and a median health care cost of 34€ for the first year after the index date. Half of the sample had more than 13 years education and approximately 80% of patients of working age were registered as employed or self-employed. Thirty percent of patients had an immigrant background while approximately 5% had two or more comorbidities. More than 75% used GP-services, 17% used hospital services, 14% used physiotherapy and 15% used chiropractors. Approximately 25% of patients used more than one health care service during the first year after a new MSD.

### Classes and characteristics

**Fig. 2:**
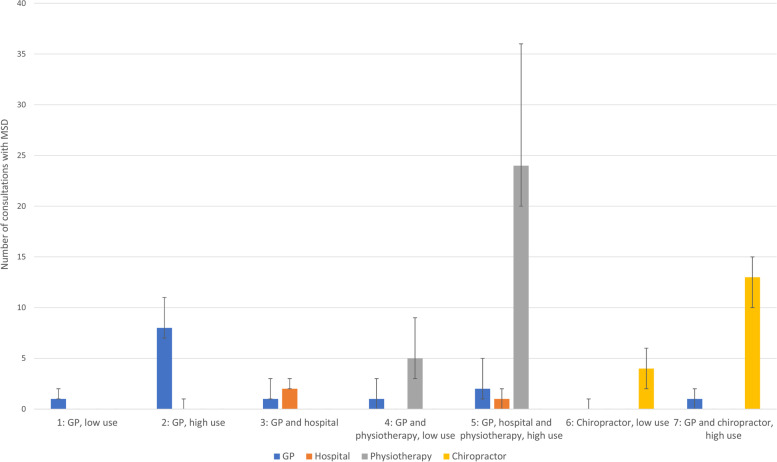
Latent classes with median and IQR of number of consultations related to MSD-diagnoses per service for GP, hospital, physiotherapy and chiropractor. Data aggregated for the first year following first consultation (index date). MSD = Musculoskeletal Disorder. GP = General Practitioner

The classes in the 7-class model can be described as 1: GP, low use; 2: GP, high use; 3: GP and hospital; 4: GP and physiotherapy, low use; 5: GP, hospital and physiotherapy, high use; 6: Chiropractor, low use; 7: GP and chiropractor, high use. A bar graph illustrating the classes with median and IQR number of consultations with MSD-diagnoses per service are presented in Fig. 2. Additional information on health care use are presented in Table 2.

**Table 2:** Latent classes and health care use

Class number and name	All	1: GP, low use *N* = 135 141 (68.2%)	2: GP, high use *N* = 4 433 (2.2%)	3: GP and hospital *N* = 7 370 (3.7%)	4: GP and physiotherapy, low use *N* = 17 636 (8.9%)	5: GP, hospital and physiotherapy, high use *N* = 6 304 (3.2%)	6: Chiropractor, low use *N* = 19 818 (10.0%)	7: GP and chiropractor, high use *N* = 6 688 (3.4%)
Used more than one health care service	27.0%	10.0%	58.8%	79.7%	79.8%	89.2%	38.0%	64.7%
Used GP	77.3%	83.7%	100%	78.8%	74.8.7%	82.1%	34.8%	58.0%
Used hospital	17.2%	10.8%	45.7%	100%	24.7%	51.0%	6.9%	14.2%
Used physiotherapy	13.7%	1.0%	14.5%	1.2%	100%	100%	0.3%	18.0%
Used chiropractor	15.0%	0.9%	12.3%	2.3%	3.9%	9.3%	100%	100%

*Abbreviations*: *GP* General practitioner

The first class of first year health care use had low health care use, mostly with the GP as the only provider with a median of one consultation. This class constituted about 70% of the sample, and had demographic, socioeconomic and clinical characteristics similar to that of the total sample. This class had the lowest number of consultations, lowest cost and low levels of sick leave. This class had the lowest proportion of long-term high-cost users (2.7%) and the highest proportion of patients with no MSD-related health care cost after the first year (28.5%). Further descriptive data for all classes are shown in Table 3.

**Table 3:** Latent classes and patient characteristics

Class number and name	All	1: GP, low use *N* = 135 976 (68.6%)	2: GP, high use *N* = 4 433 (2.2%)	3: GP and hospital *N* = 7 370 (3.7%)	4: GP and physiotherapy, low use *N* = 17 636 (8.9%)	5: GP, hospital and physiotherapy, high use *N* = 6 304 (3.2%)	6: 6: Chiropractor, low use *N* = 19 818 (10.0%)	7: GP and chiropractor, high use *N* = 6 688 (3.4%)
Age (mean (SD))	43.6 (18.0)	43.6 (18.2)	45.6 (16.3)	48.7 (20.1)	44.9 (17.7)	53.0 (17.8)	38.6 (14.7)	40.6 (14.8)
Gender (female)	49.9%	48.9%	45.4%	45.7%	55.7%	55.5%	49.8%	56.9%
Income (€) (median (IQR))	31 559 (28 873)	30 068 (28 217)	30 590 (20 107)	29 256 (28 162)	34 730 (29 897)	33 967 (24 884)	37 162 (33 234)	39 375 (29 559)
Education, 13 years or less	49.9%	51.6%	69.8%	56.7%	43.2%	55.5%	38.9%	40.0%
Employed/self-employed*	78.7%	76.7%	82.5%	73.6%	81.2%	81.5%	86.1%	89.1%
Ccomorbidity^a^, 2 or more	4.7%	5.1%	4.7%	9.0%	3.6%	5.8%	1.8%	2.0%
Immigrant background	30.7%	32.8%	47.4%	22.4%	25.0%	24.1%	25.3%	22.6%
Days with sick leave first year** (median (IQR))	0 (0)	0 (0)	79 (197)	0 (18)	0 (3)	0 (83)	0 (0)	0 (8)
Any sick leave first year**	19.6%	15.2%	74.5%	37.1%	26.5%	48.1%	13.1%	28.9%
Disability pension before index	7.5%	8.0%	6.5%	13.0%	6.7%	10.4%	3.1%	3.4%
Health care cost for MSD-health contacts (€), 1^st^ year (median (IQR))	34 (102)	20 (45)	268 (337)	498 (2285)	127 (206)	838 (1537)	-^b^	-^b^
No. consultations for MSD-health contacts, 1^st^ year (median (IQR))	2 (3)	1 (1)	9 (5)	3 (3)	7 (7)	30 (17)	5 (4)	14 (6)
GP consultations 1^st^ year, other diagnoses (median (IQR))	1 (3)	1 (3)	1 (4)	1 (3)	2 (4)	2 (4)	1 (3)	2 (4)
Hospital visits 1^st^ year, other diagnoses (median (IQR))	0 (2)	0 (1)	0 (3)	3 (5)	0 (2)	1 (4)	0 (1)	0 (1)
High-cost user year 1–5 (Above 95^th^ percentile, 3 744€)	5%	2.7%	11.8%	22.6%	8.0%	28.7%	2.8%	5.3%
No MSD-related health care costs year 2–5	24.6%	28.5%	11.0%	20.7%	18.4%	10.6%	18.5%	8.9%

*Abbreviations*: *GP* General practitioner. *MSD* Musculoskeletal disorder

^*^If age between 18–63

^**^If registered as employed same year

^a^Comorbidity index based on the International Classification of Primary Care (ICPC-2) [34]

^b^Chiropractor consultations are financed differently to other services with much lower fee-for-service refund from HELFO, making direct comparison of cost with the other classes difficult. See more in text under Methods – Design and setting

Class 2 identifies a class with a high use of GP-services for MSDs. Approximately 45% had been to a hospital consultation for their MSD, 15% used physiotherapy, 12% used chiropractors, while 41% only used their GP. This class had the highest proportion of patients being on sick leave the first year and highest median number of days of sick leave the first year. Additionally, this class had the highest proportion of patients with low education, low income, and the largest proportion of patients with an immigrant background. Twelve percent belong to the long-term high-cost user group, while 11% had no MSD-related health care cost after the first year.

Class 3 used GP and hospital services for MSDs. This class had a low number of total consultations and low use of physiotherapy and chiropractors. They had the highest comorbidity and highest hospital use for other diagnoses than MSDs. This class had a high age, high proportion of male patients, low education, low income, low proportion of patients with immigrant background and the lowest employment rate among patients of working age. They also had the highest proportion of patients on permanent disability pension prior to the index date. This class had a high proportion of patients with fractures and joint/-ligament injuries and unspecified/other MSDs. They had the second highest proportion of high-cost users with 23% and a high proportion of patients with no costs after the first year with 21%.

Class 4 used a combination of GP and a low use of physiotherapy. This class had a high proportion of females, a low proportion of patients with immigrant background, high income and high education. The class had a high prevalence of neck pain and shoulder pain. Eight percent belonged to the high-cost group while 18% had no health care costs after the first year.

Class 5 were characterised by the use of GP and a high use of physiotherapy, while approximately 50% had been to hospital for their MSD. This class had the highest median cost and number of consultations, and approximately 50% had sick leave the first year. This class had the highest age, a high proportion of females, low proportion of patients with immigrant background, low education, high comorbidity and higher use of hospital services for other diagnoses. There was a high proportion of patients with fractures or joint-/ligament injuries and osteoarthritis compared to the total prevalence. Class 5 had the highest proportion of patients that are in the long-term high-cost group (29%) and the second lowest proportion of no-cost patients (11%) after the first year.

Class 6 identified patients with a low use of chiropractors. This class had the lowest use of GP with 35% and lowest use of hospital with 7%. This class were characterised by lower age, high income, high education, low comorbidity, and low proportion of patients with immigrant background. The class had a high proportion of people registered as employed and low proportion on disability pension. The class had a high prevalence of patients with neck and back pain, and these diagnoses accounted for 70%. Only 2.8% are long-term high-cost users, while 19% are no-cost users in years two through five.

In class 7, patients were characterised by high use of chiropractor and a combination with GP services. This class shares similar characteristics with class 6, but with a higher proportion of females, higher age, higher income, lower proportion of patients with immigrant background, higher employment rate and more patients with sick leave the first year. Neck and back pain accounted for 75% of diagnoses in this class. This class had 5% in the long-term high-cost user groups and the lowest proportion of patients with no MSD-related health care costs after the first year with 9%.

### Diagnoses

**Fig. 3:**
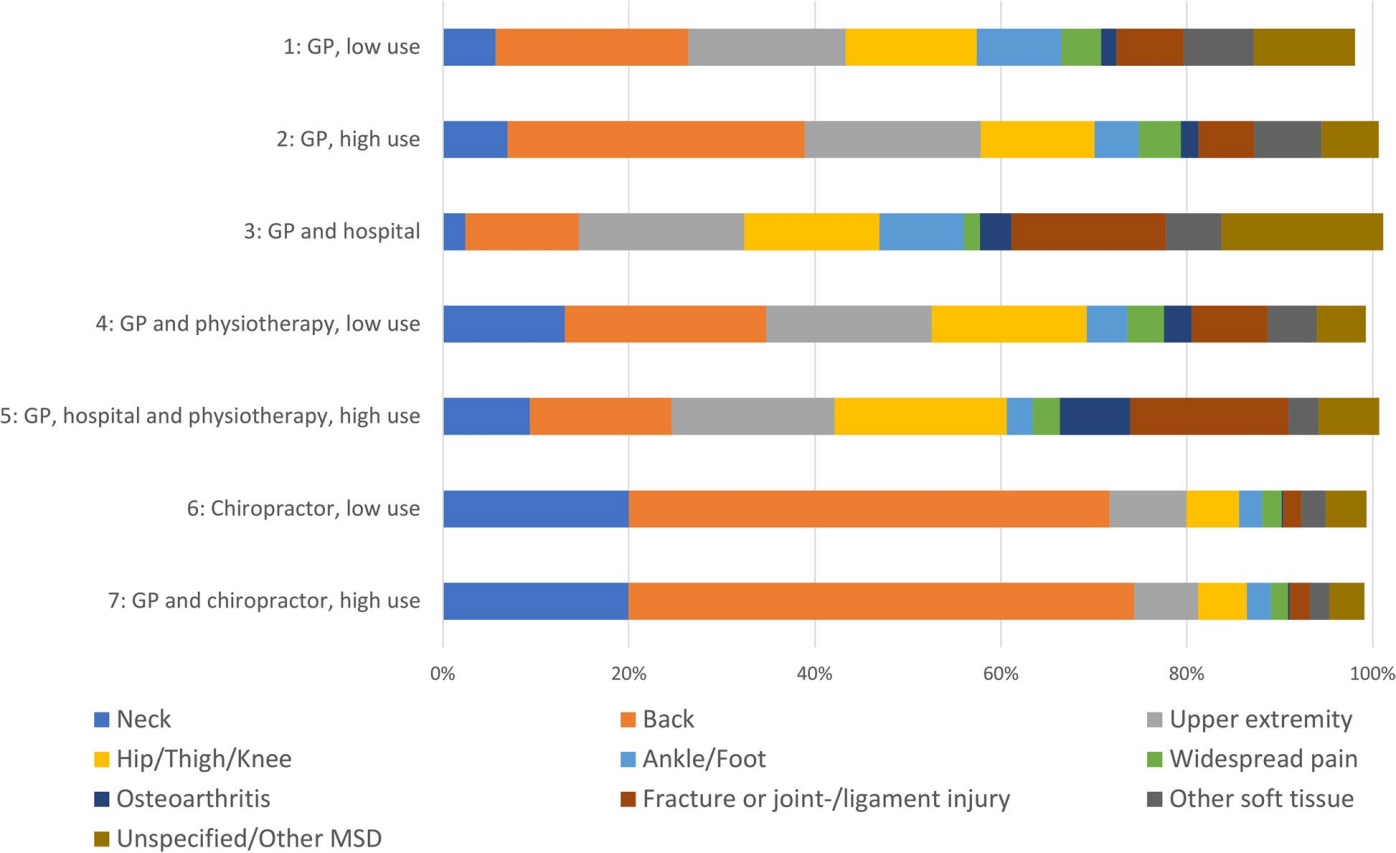
Prevalence of diagnoses for the total sample and per class. Diagnoses do not total exactly 100% as some patients have more than one index diagnose. MSD = Musculoskeletal Disorder. GP = General Practitioner

Back pain was the most prevalent diagnosis, accounting for 25% of the total sample, followed by upper extremity pain (16%), hip/thigh/knee pain (13%) and neck pain (8%). Fractures and joint-/ligament injuries were 8% of the total prevalence, ankle/foot disorders 8% and other soft tissue disorders 6%. Widespread pain and osteoarthritis accounted for 4% and 2%, respectively. Approximately 9% of the sample were diagnosed with unspecified or other MSDs. There was variation in the diagnoses distribution in the different classes, as illustrated in Fig. 3.

### Health care costs and long-term use

The total cost of health care contacts during the first year after a new MSD for the patients included was approximately 60 million Euros. Patients belonging to classes characterised by low health care utilization and treatment mainly in primary health care (classes 1, 4 and 6), accounted for 87.5% of the patient population. These classes accounted for 37% of the total costs and 54% of all consultations. The remaining 12.5% belonged to classes characterised either by more hospital use, high use of one service or higher combinations of services (classes 2, 3, 5 and 7). These 12.5% accounted for 63% of costs and 46% of consultations.

Approximately one-third of all costs were related to the first year after the index date, while two-thirds were related to health care contacts in the following four years. DRG-costs in hospital were responsible for 74% of total costs in the first five years, physiotherapy consultations for 12% and GP-consultations for 10%. Patients above the 99^th^ percentile of health care costs in the first five years (more than 12 714 €) accounted for 23% of the total costs, those above the 95^th^ percentile (3 744 €) for 58% of costs and those above the 90^th^ percentile (1 743 €) for 75% of costs. The 95^th^ percentile was used as a cut-off representing high-cost patients. For these patients, DRG-related costs in hospital accounted for 89% of total costs, physiotherapy consultations for 8%, and GP-consultations for 3%.

We also found that approximately 25% of patients had no MSD related costs after the first year. After the second year, the proportion that did not have any costs the following years increased to 33%. The proportions of patients that are high-cost patients (above 95^th^ percentile) and no-cost patients after the first year for each class are shown in Table 3.

### Subgroup comparison

In class 2: GP, high use and class 5: GP, hospital and physiotherapy, high use, there were close to 50% of patients that used hospital services while the other half did not. Therefore, it is relevant to perform a subgroup comparison of these groups to assess whether characteristics within the group differ based on hospital use. The subgroup without hospital use in class 2 had median cost of 171€ and the group with hospital use had 460€. The subgroups were largely similar based on patient characteristics but patients with hospital use had a higher median of sick leave days and a higher proportion of osteoarthritis and knee pain diagnoses. In the subgroup without hospital use, 5% were classified as long-term high-cost users and 13% were in the no-cost group. For the subgroup with hospital use 20% were high-cost users and 9% had no MSD-related health care costs in year two to five.

For class 5: GP, hospital and physiotherapy, high use, the subgroup without hospital use had a median cost of 532€ and the group with hospital use had a median cost of 1 917€. The subgroup without hospital use had a higher proportion of females compared to the group with hospital. The subgroup with hospital use had higher sick leave. The no-hospital subgroup had more patients with neck, back and widespread pain, while the hospital subgroup had more patients with fracture or joint-/ligament injuries and unspecified/other MSDs. The subgroup with hospital use had 46% of patients in the long-term high-cost group while the group without hospital use had 11%. The proportion of patients with no future health care costs were similar in the two groups, with 10% for the group with hospital use and 11% for the group without hospital use. Full comparison of the subgroups is provided in the supplementary (supplementary 2).

## Discussion

We identified seven distinct classes based on the combinations of health care services utilized the first year after receiving an MSD-diagnosis. The classes varied substantially in their use of the different services and in the total resource consumption. The classes with the lowest use had a median of only one consultation while the class with highest use had a median of 30 consultations. The classes had large differences in diagnoses, and demographic and socioeconomic factors, which indicates that these factors are important for how patients use health care services in their management of MSD.

### Impact of demographic and socio-economic factors on health care use

Patients with lower socioeconomic status have a higher burden of chronic diseases, higher risk of poor outcomes and poorer general and musculoskeletal health [16–18]. Socioeconomic status has the potential for influencing health status through multiple pathways, such as occupational position, reduced occupational hazard, higher income, healthier lifestyle, and higher health literacy [16]. The same mechanisms are thought to also influence musculoskeletal health as well [21]. Poor health and low socioeconomic status may have a self-reinforcing mechanism, where poor health limits employment possibilities, which leads to economic deprivation, psychological distress and subsequently worse health outcomes [20]. Additionally, patients with lower education might have more difficulties in self-managing their health conditions [20, 24, 48]. These previously identified mechanisms are in line with our findings where classes with higher health care use generally included more patients with lower socioeconomic status. This is supported by previous studies on health care use from other countries which demonstrate that higher health care use is associated with lower socioeconomic status for MSD [15, 46].

Ideally, the needs for health care should be the only determinant of health care use, but other factors such as access to services, social barriers, economic factors and health care system organisation also play a role [49]. These factors are also likely to influence health care utilization for MSD and may explain some of the variation in our findings. Our results showed that the class that used GP and hospital had a low proportion of patients with immigrant background compared to those that predominantly used GP services. This may indicate that these patients had lower access to specialist health care for MSD. These findings are supported by previous research, which has shown that patients with immigrant background had lower use of specialist health care in Norway [50]. For the classes that used physiotherapy, we found that these patients had a higher income and education and lower proportions of immigrant background than those that predominantly used GP services. The class with higher physiotherapy use and more combinations with other services also had higher age, more comorbidity and lower education compared to the class with low physiotherapy use. This might indicate that for patients with access to physiotherapy, there is a higher use among patients with higher risk of poor outcomes, but that there is a lower access to the service for patients with lower socioeconomic status and immigrant background. This is supported by previous findings that show socioeconomic inequalities in use of physiotherapy in Norway [51]. The patients who use chiropractor has a different demographic and socioeconomic profile compared to the other classes, which is expected given the significant difference in how this service is financed.

It is likely that both access to services, clinical and non-clinical factors influence how patients uses health care services for MSD. It is therefore particularly interesting that patients in class 2: GP, high use had a relatively low use of other health care services than GP and a low health care cost compared to other high-use classes. They also had the lowest education, lowest income and highest proportion of immigrants. As the class simultaneously had a high proportion of patients on sick leave and a high proportion with future health care costs, this finding does not seem to be explained by low severity or low disability related to the MSD. A possible interpretation could be that this identifies a class with high risk of poorer outcomes but lower access to health care services for MSD, other than GP services. It is unknown whether the low use of other health care services is due to lack of referral to other services, the patient does not wish to use other health services, or lack of access due to other barriers. This indicates a gap between risk of future negative outcomes and the access to health services that should be considered when planning future care.

### High-cost patients and long-term use for MSD

There is an ongoing discussion on resource use in health care, where the increasing prevalence of MSDs [2] and limited economic resources makes it important to prioritize patients with the highest needs and the most cost-effective health care services [52]. Our study demonstrates that a small proportion of patients are responsible for a large proportion of the total resource use. Hospital care accounts for most of the total costs in our data, and the majority of hospital costs for MSDs have been shown to be related to orthopaedic surgical interventions [4]. Our results show that there is a higher prevalence of specific diagnoses such as fractures or joint-/ligament injuries and osteoarthritis in classes with hospital use. However, there is still a high proportion of patients without specific diagnoses, suggesting that other factors than diagnoses are also important for determining who are referred to specialist health care. It may also be the case that the ICPC-2 diagnoses codes are not sufficiently detailed to capture clinically important variation in diagnoses. Our study indicates that reducing primary health care use is unlikely to produce substantial cost reduction and approaches to reduce specialist health care costs would have the largest effect on reducing total health care costs. From a cost perspective, it would be most effective if patients with a high health care need, not related to specific clinical factors, are helped in primary health care services while patients with specific diagnoses where treatment in specialist health care is superior, are prioritised in specialist health care. The majority of total costs are concentrated among a low proportion of patients, highlighting the need for more research on these patients and their treatment.

Our findings also show that many patients require long-term health care use. Especially in classes with high initial use there is a large proportion of patients that continues to use the health care services for MSD after the first year. Many patients do not fit the model where they have a short-term health care use and then do not seek further help, and previous research shows that it is common for patients with MSDs to have both ongoing pain, episodic pain and recurrence of pain [53–57]. This indicates that it is necessary to plan for more long-term health care use rather than just for short-term use when organising MSD-care, and to acknowledge that many patients have ongoing, episodic or recurrent pain that they will seek help for several times. This challenges the suggested model of standardising care pathways, where patients often follow a treatment process for a predefined time-period and then are discharged [58, 59]. There is currently a lack of data on what is the most effective approach for managing patients with increased risk of poor outcomes and high health care costs.

### Strengths and limitations

The use of data registers that automatically include the entire population and the ability to link registers on an individual level provides a unique dataset with complete information on public primary and specialist health care use, welfare use, demographic, and socioeconomic factors. This makes it possible to create a comprehensive cohort without risking problems with selection bias that could influence the validity of the findings [60]. Thus, the study gives a more accurate overview over real life clinical practice than what is possible to achieve with other study designs, such as surveys and selected cohorts [61]. Registering data in both primary and specialist health care is necessary to receive reimbursement for both primary health care clinicians and hospitals, increasing the likelihood that the health care databases are complete. The database on welfare systems is also connected to pay-outs from Labour and Welfare Administration (NAV).

Another strength is that we compared our findings to latent classes in a cohort included from 2018–2019 (supplementary). The results showed very similar classes for the 2013–2015 cohort and 2018–2019 cohort, indicating that the dynamics of health care use for MSDs have not changed, and that our findings are stable over time. These findings represent health care utilisation before the COVID19-pandemic, and it is uncertain whether the pandemic changed the dynamic of health care use for MSDs.

This study has used primary care diagnoses from GP and physiotherapy consultations, which uses the International Classification of Primary Care, 2nd version (ICPC-2). ICPC-2 has shown to have good validity for GP-consultations but less so for other registrations, such as simple contacts and prescription writing [35]. Our study only uses data from consultations, indicating that the validity of this approach is good. However, there are some challenges for using ICPC-2 diagnoses. Previous research show that several problems or complaints are brought up in most GP visits, which increases the uncertainty of what diagnosis is the correct one for each consultation [62]. Moreover, there is no previous research on the validity of ICPC-2 codes in physiotherapy or for chiropractors. Additionally, most patients with MSDs are found to have multiple pain sites, which could make it challenging to select a diagnosis from a body region-based code system [63]. Some conditions, such as fibromyalgia, often have a long time from initial contact to diagnosis [64, 65]. This indicates that our approach by selecting the first registered diagnosis is likely to underestimate the true prevalence of some conditions. Another limitation is that our definition of widespread pain is based on the ICPC-2 code for widespread pain/fibromyalgia. This is probably a more narrow definition of widespread pain compared to other studies where it is defined by reporting multiple pain sites [66, 67]. This could lead to an underestimation of this diagnosis category in our study.

Differing coding behaviour between clinicians and professions, and different software between clinics might also challenge the validity of diagnosis codes [68], leading to increased risk for the MSD-diagnosis to change multiple times during a follow-up period, due to clinician behaviour rather than changes in symptoms and severity. To account for this, we have included all MSDs as one broad category, and not categorized on individual codes, as this would most likely reduce the accuracy further. The index diagnosis and their distribution along the classes are presented in the results chapter to make the classes understandable from a clinical context.

The diagnosis codes give no information on severity or patient reported measures. It is likely that that disease severity and patient reported measures such as pain and function could help to further explain health care use. Previous research has shown that patients treated in specialist and primary health care for neck and back pain differ in both clinical, demographic and socioeconomic factors [69].

It is a limitation that we only have access to reimbursement costs and therefore cannot calculate the total cost including the patients’ out-of-pocket expenses. This means that our reported health care costs underestimates the true cost of consultations for GP, physiotherapy and hospital outpatient services, as these services are partially financed from out-of-pocket expenses. The reimbursement costs and out-of-pocket expenses cover approximately half each of the total cost for all these services. The chiropractor services are financed mostly by out-of-pocket expenses, indicating that the cost data we have access to are clearly underestimated, as acknowledged previously.

Another limitation is that we can only include patients that use the public health care system. There is an ambition in Norway that all parts of the population should have equal access to public high-quality health care services [50] and public health care systems account for most health consultations in both primary and secondary healthcare. Still, the use of private health care financed through insurance or self-financed for MSD are increasing and in 2021 approximately 13% of the population had private health insurance [70]. Even though most health care consultations are financed through the public health care system, a substantial amount of patients use private health care. As private health care is not included in registers, we have no information on this health care use.

### Implications

This study has important implications, as it provides novel insights into the combination of health care services for MSDs and describes different patient characteristics between classes. This is valuable as it shows large variation in how patients with MSDs use the health care services, and the identification of these patterns can aid in the planning of future care, reduce disparities and improve health outcomes for patients. The identified classes showed considerable differences in demographic and socioeconomic factors, highlighting the significance of these factors for how patients use health care services in managing their MSDs. Furthermore, the study provides insight into the distribution of health care resources, with a relatively low proportion of patients responsible for a large proportion of total resource use. This study contributes to the understanding of clinical pathways and health care use in current practice, which is vital when discussing future organisational changes to health care services to reduce unwarranted variation and health care costs.

The study also highlights some challenges that needs to be accounted for in the planning of MSD-care. These include the need for long-term health care use, equal access to health care services that accounts for patients with low socioeconomic status and immigrant background, and the support of health care services that can potentially reduce high costs in hospital. It might be useful to focus on the patients that accounts for the highest proportions of health care resources when researching cost-effective care for MSDs and clinical outcomes for these patients, as these patients account for the majority of total costs and are overrepresented in long-term health care use. Future guidelines and models might be more useful if they provide guidance on best management of patients with high risk of poor outcomes and high resource use, irrespective of diagnosis or region, while also taking into account that many patients have a long-term health care use.

Direct comparisons between countries is difficult due to large differences in health care organisation, welfare systems and social structures. We believe that the classes in our study may be generalizable to other countries with similar health care organisation and structure. The volume of the different classes are likely to differ between countries due to organisation, access to services and financial barriers.

## Conclusion

We have identified seven distinct classes of combinations of health care service use during the first year after a new MSD. This novel finding provides information on what combinations of services are observed in the patient population, how frequent they are and shows the large variation in service use. The 12.5% belonging to the classes with higher use account for more than 60% of first year costs, close to 50% of first year consultations and have a large variation in how they use the health care services. Only a quarter of all patients have no MSD-related health care cost after the first year, suggesting that most patients have either repetitive or ongoing use of health care services for MSDs over several years. It is uncertain whether the observed variation in use of services indicate inefficiencies in health care delivery or variations in patient-need. Given the large differences in demographic and socioeconomic variables across the classes, it is plausible that the variation may be due to differences in patient-need rather than inefficiencies in care delivery. More research is required to determine whether the observed variations are due to inefficiencies in health care delivery.

### Supplementary Information

Additional file 1:** Supplementary 1. **Statistics and descriptions for Latent Class Analysis models.

Additional file 2: **Supplementary 2**. Subgroup comparison for class 2 and 5.

Additional file 3: **Supplementary 3**. Health care use in 2018-2019 cohort.

Addtional file 4: **Supplementary 4**. Sensitivity analysis.

## Data Availability

The datasets used in the current study are based on national registries and are not publicly available. Access to pseudonymized data from the national registries are only granted through application to the Norwegian Centre for Research Data and Regional Committees for Medical and Health Research Ethics. Other data and materials can be delivered by the corresponding author on reasonable request.

## Abbreviations

MSD: Musculoskeletal disorder
INOREG: INnovations in use Of REGistry data
STROBE: Strengthening the Reporting of Observational Studies in Epidemiology
RECORD: REporting of studies Conducted using Observational Routinely-collected Data
HELFO: The Norwegian Health Economics Administration
KUHR: The Control and reimbursement of health care claims
DRG: Nordic diagnosis-related groups
NPR: Norwegian Patient Registry
FD-Trygd: Statistics Norway's historical events database
LCA: Latent Class Analysis
GP: General practitioners
ICPC-2: International Classification of Primary Care, 2nd version (ICPC-2)
