# Supplementary material for: Combination of health care service use and the relation to demographic and socioeconomic factors for patients with musculoskeletal disorders: a descriptive cohort study
Source: BMC Health Serv Res. 2023 Aug 14;23:858. doi: 10.1186/s12913-023-09852-3 (PMC10426198; doi:10.1186/s12913-023-09852-3)
Supplement: Supplementary file 1 — Additional file 1: Supplementary 1. Statistics and descriptions for Latent Class Analysis models. [file 12913_2023_9852_MOESM1_ESM.docx]

**Supplementary 1: Statistics and descriptions for Latent Class Analysis models**

| Groups | Log-likelihood | % change  Log-likelihood | BIC | % change BIC | AIC | % change AIC | Average Posterior Probability  (min-max) | Likelihood-ratio test |
| --- | --- | --- | --- | --- | --- | --- | --- | --- |
| 1 | -1775136 |  | 3550280 |  | 3550321 |  |  |  |
| 2 | -1208102 | 31.9 | 2416221 | 31.9 | 2416221 | 31.9 | .99-.99 | <.001 |
| 3 | -965347 | 20.1 | 1930723 | 20.1 | 1930865 | 20.1 | .98-.99 | <.001 |
| 4 | -893154 | 7.5 | 1786346 | 7.5 | 1786539 | 7.5 | .97-.98 | <.001 |
| 5 | -866597 | 3.0 | 1733487 | 3.0 | 1733487 | 3.0 | .88-.99 | <.001 |
| 6 | -858251 | 1.0 | 1716856 | 1.0 | 1716856 | 1.0 | .83-.98 | <.001 |
| **7** | **-840211** | **2.1** | **1680837** | **2.1** | **1680837** | **2.1** | **.83-.98** | **<.001** |
| 8 | -825356 | 1.8 | 1651187 | 1.8 | 1650789 | 1.8 | .82-.98 | <.001 |
| 9 | -817979 | 0.9 | 1636495 | 0.9 | 1636046 | 0.9 | .82-.97 | <.001 |
| 10 | -805013 | 1.6 | 1610124 | 1.6 | 1610124 | 1.6 | .82-.96 | <.001 |
| 11 | -801828 | 0.4 | 1603763 | 0.4 | 1603763 | 0.4 | .67-.97 | <.001 |
| 12 | -797359 | 0.6 | 1594836 | 0.6 | 1594836 | 0.6 | .81-.97 | <.001 |
| 13 | -795766 | 0.2 | 1591659 | 0.2 | 1591659 | 0.2 | .82-.96 | <.001 |

Description, median number of consultations for GP, hospital, physiotherapy and chiropractor (Presented as x/x/x/x) and proportions of classes in LCA-models from 1 to 13 classes. New classes compared to classes in the previous models are highlighted with asterix*

|  | Description ‘ | Median consultations for GP/hospital/physiotherapy/ chiropractor | Proportions |
| --- | --- | --- | --- |
| *1* |  |  |  |
| 2 | GP use  GP and physiotherapy | 1/0/0/0  2/0/9/0 | 89.8  10.2 |
| 3 | GP use  GP and physiotherapy  Chiropractor use* | 1/0/0/0  2/0/10/0  0/0/0/5 | 77.8  9.9  12.4 |
| 4 | GP use  GP and physiotherapy, low use  GP and physiotherapy, high use*  Chiropractor use | 1/0/0/0  1/0/5/0  2/0/24/5  0/0/0/5 | 74.9  9.4  3.2  12.4 |
| 5 | GP use  GP and hospital  GP and physiotherapy  GP, hospital and physiotherapy*  Chiropractor use | 1/0/0/0  6/2/0/0  1/0/5/0  2/1/24/0  0/0/0/5 | 71.5  3.9  9.0  3.3  12.4 |
| *6* | GP, low use  GP, high use*  GP and hospital  GP and physio, low use  GP and physio, high use  Chiropractor use | 1/0/0/0  8/0/0/0  1/1/0/0  1/0/5/0  2/0/24/0  0/0/0/5 | 69.6  2.3  3.7  8.9  3.3  12.2 |
| 7 | GP, low use  GP, high use  GP and hospital  GP and physio, low use  GP and physio, high use  Chiropractor, low use  GP and chiropractor, high use* | 1/0/0/0  8/0/0/0  1/1/0/0  1/0/5/0  2/0/24/0  0/0/0/5  1/0/0/13 | 68.6  2.2  3.7  8.9  3.2  10.0  3.4 |
| 8 | GP, low use  GP, high use  GP and hospital  GP and physio, low use  GP and physio, high use  GP, physio and chiropractor*  Chiropractor, low use  GP and chiropractor, high use | 1/0/0/0  8/0/0/0  1/1/0/0  1/0/5/0  2/0/25/0  2/0/6/6  0/0/0/3  1/0/0/12 | 68.2  2.2  3.7  8.7  3.0  1.0  9.6  3.7 |
| 9 | GP, low use  GP, high use  GP and hospital  GP, hospital and physio, high use GP*  GP, hospital and physio, high use physio*  GP and physio, low use  GP and physio, high use  Chiropractor, low use  GP and chiropractor, high use | 1/0/0/0  8/0/0/0  1/2/0/0  6/2/11/0  3/1/40/0  1/0/4/0  2/0/25/0  0/0/0/4  1/0/0/12 | 68.2  2.1  3.7  1.5  1.2  8.7  3.0  9.9  3.7 |
| 10 | GP, low use  GP, high use  GP and hospital  GP, hospital and physio  GP and physio, low use  GP and physio, medium use*  GP and physio, high use  GP, physio and chiropractor  Chiropractor, low use  GP and chiropractor, high use | 1/0/0/0  8/0/0/0  1/1/0/0  7/1/9/0  1/0/4/0  1/0/19/0  3/0/43/0  2/0/6/6  0/0/0/3  1/0/0/12 | 68.1  2.1  3.7  1.3  7.0  2.7  1.0  0.9  9.6  3.6 |
| 11 | GP, low use  GP, high use  GP and hospital  GP, hospital and physio, low use physio*  GP, hospital and physio, medium use physio*  GP, hospital and physio, high use physio  GP and physio, low use  GP and physio, high use  GP, physio and chiropractor  Chiropractor, low use  GP and chiropractor, high use | 1/0/0/0  7/0/0/0  1/2/0/0  5/1/7/0  6/2/25/0  2/1/47/0  1/0/4/0  1/0/17/6  2/0/6/6  0/0/0/3  1/0/0/12 | 67.4  2.2  3.7  1.5  0.8  0.8  7.0  2.6  0.9  9.6  3.6 |
| 12 | GP, low use  GP, high use  GP and hospital  GP, hospital and physio, low use physio  GP, hospital and physio, medium use physio  GP, hospital and physio, high use physio  GP and physio, low use  GP and physio, high use  GP, physio and chiropractor  GP and chiropractor, medium use*  Chiropractor, low use  GP and chiropractor, high use | 1/0/0/0  7/0/0/0  1/2/0/0  5/1/7/0  6/2/25/0  2/1/47/0  1/0/4/0  1/0/17/6  2/0/7/6  4/0/0/6  0/0/0/3  1/0/0/12 | 67.0  2.6  3.6  1.5  0.8  0.8  7.0  2.6  0.9  1.0  9.4  3.0 |
| 13 | GP, low use  GP, high use  GP and hospital, low use  GP and hospital, high use hospital*  GP, hospital and physio, low use physio  GP, hospital and physio, medium use physio  GP, hospital and physio, high use physio  GP and physio, low use  GP and physio, high use  GP, physio and chiropractor  GP and chiropractor, medium use  Chiropractor, low use  Chiropractor, high use | 1/0/0/0  7/0/0/0  1/2/0/0  2/9/0/0  6/1/7/0  6/2/26/0  2/1/47/0  1/0/4/0  1/0/17/0  1/0/7/6  4/0/0/6  0/0/0/3  0/0/0/12 | 67.1  2.5  3.6  0.1  1.5  0.8  0.8  6.8  2.5  0.9  0.9  9.4  3.0 |

‘ Class descriptions are based on how the class is distinct from the other classes in the same model. The classes are first described by the type of health care services that are used. When there are more than one class with the same service use, it is further described by being low or high use of primary care services relatively to the other class in the same model. Only one class is defined by “high use hospital”, and is found in model 13.
