# Supplementary material for: Combination of health care service use and the relation to demographic and socioeconomic factors for patients with musculoskeletal disorders: a descriptive cohort study
Source: BMC Health Serv Res. 2023 Aug 14;23:858. doi: 10.1186/s12913-023-09852-3 (PMC10426198; doi:10.1186/s12913-023-09852-3)

**Supplementary 2: Subgroup comparison for class 2 and 5.**

| Class number and name | 2: High use GP | Without hospital use | With hospital use | 5: GP, hospital and physiotherapy, high use | Without hospital use | With hospital use |
| --- | --- | --- | --- | --- | --- | --- |
| Total health care cost (Euro) | 268 (337) | 171 (167) | 459 (663) | 838 (1537) | 532 (459) | 1917 (4120) |
| Age (mean (SD)) | 45.6 (16.3) | 44.9 (16.5) | 46.5 (16.0) | 53.0 (17.8) | 53.4 (18.0) | 52.6 (17.6) |
| Gender (female) | 45.4% | 47.7% | 42.7% | 55.5% | 60.8% | 50.5% |
| Large/high income municipality | 86.3% | 88.2% | 84.0% | 84.8% | 87.4% | 82.3% |
| Income below 400 000 NOK | 62.2% | 63.2% | 61.0% | 52.8% | 53.9% | 51.8% |
| Education, 13 years or less | 69.8% | 69.9% | 69.6% | 55.5% | 54.5% | 56.4% |
| Employed/self-employed* | 82.5% | 83.6% | 81.1% | 81.5% | 79.0% | 83.7% |
| ICPC comorbidity index, 2 or more | 4.7% | 4.5% | 4.9% | 5.8% | 6.1% | 5.6% |
| Immigrant background | 47.4% | 50.3% | 54.0% | 24.1% | 23.5% | 24.8% |
| Days with sick leave first year (median (IQR))** | 79 (197) | 72 (179) | 88.5 (218.5) | 0 (83) | 0 (43) | 20 (119) |
| Proportion with any sick leave first year** | 74.5% | 74% | 75% | 48.1% | 36.1% | 58.7% |
| Proportion on permanent disability pension before index | 6.5% | 6.0% | 7.1% | 10.4% | 11.6% | 9.3% |
| High-cost user year 1-5 (Above 95^th^ percentile) | 11.8% | 5.0% | 20.1% | 28.7% | 11.3% | 46.0% |
| No MSD-related health care costs year 2-5 | 11.0% | 12.6% | 9.0% | 10.6% | 11.0% | 10.1% |

Supplementary figure: Subgroup comparison of diagnosis prevalence for class 2 and 5. Diagnosis do not add up to exactly 100% as some patients have more than one index diagnosis.


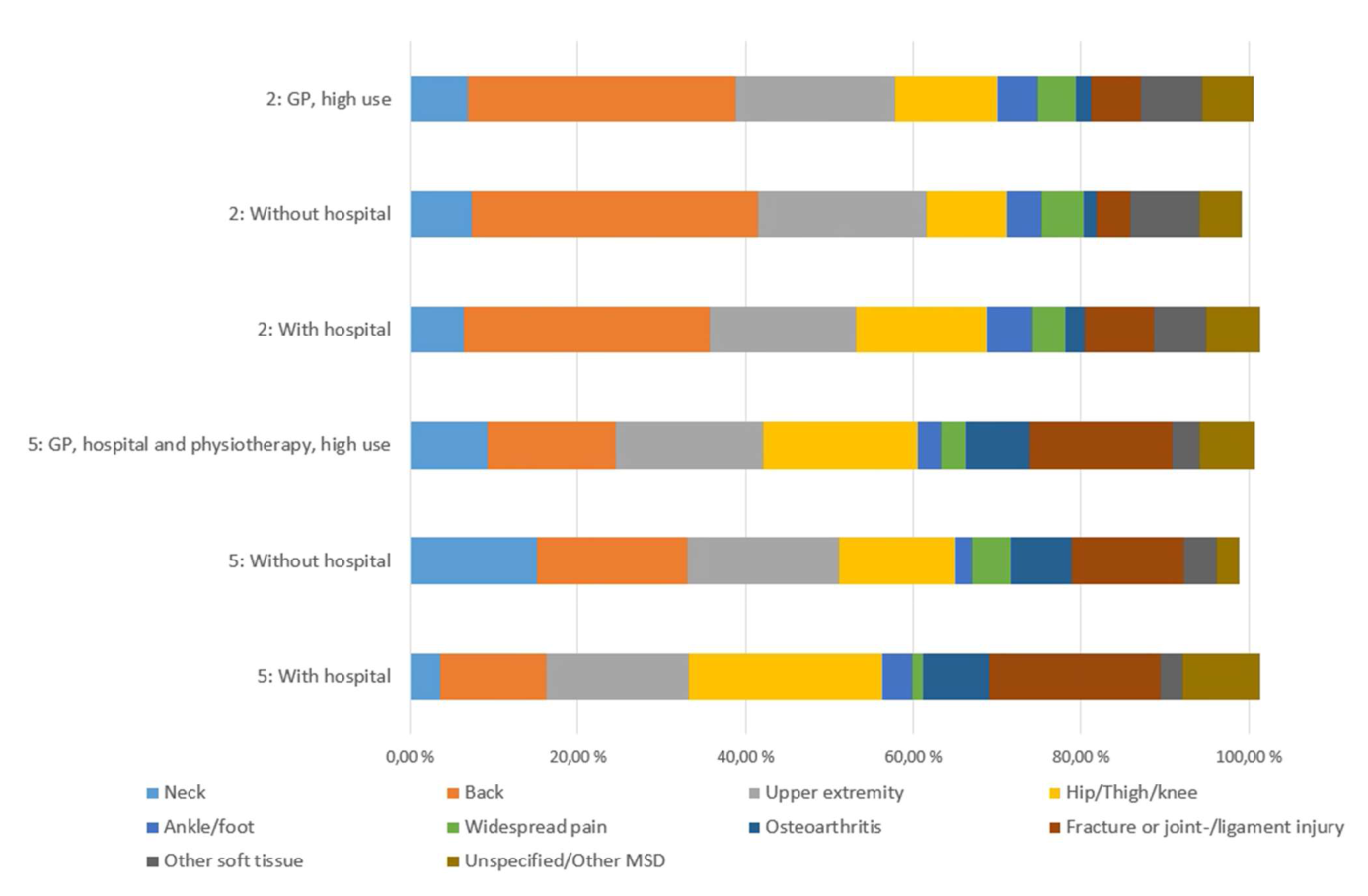

Supplement: Supplementary file 2 — Additional file 2: Supplementary 2. Subgroup comparison for class 2 and 5. [file 12913_2023_9852_MOESM2_ESM.docx]
