# Supplementary material for: Combination of health care service use and the relation to demographic and socioeconomic factors for patients with musculoskeletal disorders: a descriptive cohort study
Source: BMC Health Serv Res. 2023 Aug 14;23:858. doi: 10.1186/s12913-023-09852-3 (PMC10426198; doi:10.1186/s12913-023-09852-3)
Supplement: Supplementary file 3 — Additional file 3: Supplementary 3. Health care use in 2018-2019 cohort. [file 12913_2023_9852_MOESM3_ESM.docx]

| Class number and name | All | 1: Low use, GP only | 2: High use GP | 3: GP and hospital | 4: GP and physiotherapy, low use | 5: GP and physiotherapy, high use | 6: Low use chiropractor | 7: GP and chiropractor, high use |
| --- | --- | --- | --- | --- | --- | --- | --- | --- |
| N (%) | 133 754 (100%) | 91 050 (68.1%) | 3 182 (2.4%) | 5 866 (4.4%) | 12 072 (9.0%) | 3 314 (2.5%) | 15 067 (11.3%) | 3 203 (2.4%) |
| GP consultations | 1 (2) | 1 (1) | 9 (4) | 1 (2) | 2 (1) | 2 (4) | 0 (1) | 1 (2) |
| Hospital consultations | 0 (0) | 0 (0) | 1 (2) | 2 (1) | 0 (0) | 0 (2) | 0 (0) | 0 (0) |
| Physiotherapy consultations | 0 (0) | 0 (0) | 0 (3) | 0 (0) | 4 (4) | 24 (16) | 0 (0) | 0 (0) |
| Chiropractor consultations | 0 (0) | 0 (0) | 0 (0) | 0 (0) | 0 (0) | 0 (0) | 4 (4) | 14 (7) |
| Proportion used more than one health care service | 27.6% | 11.2% | 78.3% | 83.0% | 70.9% | 83.8% | 39.4% | 63.4% |
| Proportion used GP-service | 76.2% | 83.9% | 99.9% | 81.8% | 64.9% | 76.9% | 36.0% | 55.0% |
| Proportion used specialist health care | 18.0% | 11.8% | 55.1% | 100% | 20.3% | 46.8% | 7.8% | 16.1% |
| Proportion used physiotherapy | 14.2% | 1.4% | 45.2% | 2.3% | 100% | 100% | 20.3% | 0.9% |
| Proportion used chiropractor | 15.5% | 1.0% | 18.4% | 1.8% | 4.7% | 8.7% | 100% | 100% |
| 1^st^ year health care cost for MSD-health contacts | 432 (1340) | 268 (580) | 4977 (7599) | 9431 (25564) | 1349 (1959) | 9323 (12416) | 474 (476) | 1575 (1510) |
| 1^st^ year no. consultations for MSD-health contacts | 2 (3) | 1 (1) | 11 (6) | 3 (3) | 6 (5) | 29 (18) | 5 (4) | 16 (8) |
| GP consultations, other diagnosis | 1 (4) | 1 (3) | 1 (4) | 1 (4) | 2 (4) | 2 (3) | 1 (3) | 2 (4) |
| Hospital consultations, other diagnosis | 0 (2) | 0 (1) | 1 (3) | 3 (5) | 0 (2) | 1 (4) | 0 (1) | 0 (1) |

**Supplementary 3 – Health care use in 2018-2019 cohort**
