# Supplementary material for: Combination of health care service use and the relation to demographic and socioeconomic factors for patients with musculoskeletal disorders: a descriptive cohort study
Source: BMC Health Serv Res. 2023 Aug 14;23:858. doi: 10.1186/s12913-023-09852-3 (PMC10426198; doi:10.1186/s12913-023-09852-3)
Supplement: Supplementary file 4 — Addtional file 4: Supplementary 4. Sensitivity analysis. [file 12913_2023_9852_MOESM4_ESM.docx]

**Supplementary 4: Sensitivity analysis**

**Cross tabulation of LCA-class and high-cost user above 99^th^ percentile and 90^th^ percentile year 1-5, 99^th^, 95^th^ and 90^th^ percentile year 2-5 and no cost users after year 2**

| Class number and name | All | 1: Low use, GP only | 2: High use GP | 3: GP and hospital | 4: GP and physiotherapy, low use | 5: GP, hospital and physiotherapy, high use | 6: Low use chiropractor | 7: GP and chiropractor, high use |
| --- | --- | --- | --- | --- | --- | --- | --- | --- |
| High-cost user year 1-5 (Above 99^th^ percentile, 12 603€) | 1% | 0.2% | 1.9% | 9.0% | 1.7% | 10.7% | 0.1%  ^a^ | 0.5%  ^a^ |
| High-cost user year 1-5 (Above 90^th^ percentile, 1 728€ ) | 10% | 5.9% | 24.3% | 43.0% | 15.9% | 49.3% | 5.8%  ^a^ | 10.1%  ^a^ |
| High-cost user year 2-5 (Above 99^th^ percentile 10 657€) | 1% | 0.7% | 2.1% | 2.4% | 1.6% | 4.7% | 0.6%  ^a^ | 1.0%  ^a^ |
| High-cost user year 2-5 (Above 95^th^ percentile, 2 559€) | 5% | 3.9% | 10.1% | 11.0% | 7.0% | 16.1% | 3.9%  ^a^ | 5.7%  ^a^ |
| High-cost user year 2-5 (Above 90^th^ percentile, 883€) | 10% | 7.9% | 23.1% | 19.1% | 14.2% | 31.2% | 7.7%  ^a^ | 11.5%  ^a^ |
| No MSD-related health care costs year 3-5 | 33.1% | 36.7% | 21.4% | 30.1% | 27.7% | 20.4% | 26.6% | 17.7% |

^a^ Chiropractor consultations are financed differently to other services with much lower fee-for-service refund from HELFO, making it difficult to compare expenses with the other classes. See more in text under Methods – Design and setting.
